# Continuous adaptation of conversation aids for uterine fibroids treatment options in a four-year multi-center implementation project

**DOI:** 10.1186/s12911-024-02637-6

**Published:** 2024-09-30

**Authors:** Danielle Schubbe, Marie-Anne Durand, Rachel C. Forcino, Jaclyn Engel, Marisa Tomaino, Monica Adams-Foster, Carla Bacon, Carrie Cahill Mulligan, Sateria Venable, Tina Foster, Paul J. Barr, Raymond M. Anchan, Shannon Laughlin-Tommaso, Anne Lindholm, Maya Seshan, Rossella M. Gargiulo, Patricia Stephenson, Karen George, Mobolaji Ajao, Tessa Madden, Erika Banks, Antonio R. Gargiulo, James O’Malley, Maria van den Muijsenbergh, Johanna W. M. Aarts, Glyn Elwyn

**Affiliations:** 1 The Dartmouth Institute for Health Policy and Clinical Practice, Geisel School of Medicine at Dartmouth College, One Medical Center Drive, Williamson Translational Research Building Level 5, Lebanon, NH 03756 USA; 2 Unisanté, Centre universitaire de médecine générale et santé publique, Rue du Bugnon 44, Lausanne, CH-1011 Switzerland; 3 Department of Population Health, University of Kansas School of Medicine, Kansas City, KS USA; 4 Rutgers Institute for Nicotine & Tobacco Studies, New Brunswick, NJ USA; 5 National Uterine Fibroids Foundation, Colorado Springs, CO 80909 USA; 6 Fibroid Foundation, 10319 Westlake Dr, Suite 102, Bethesda, MD 20817 USA; 7 Department of Obstetrics, Gynecology & Reproductive Biology, Brigham and Women’s Hospital, Harvard Medical School, Boston, MA USA; 8 Department of Obstetrics and Gynecology, Mayo Clinic, Rochester, MN USA; 9 Department of Obstetrics and Gynecology, Larner College of Medicine, University of Vermont, Burlington, VT USA; 10 Department of Surgery, Division of Public Health Sciences, Washington University School of Medicine, St. Louis, MO USA; 11 Department of Obstetrics and Gynecology and Women’s Health, Albert Einstein College of Medicine, Montefiore Medical Center, Bronx, NY USA; 12 Department of Primary and Community Care, Radboud University Medical Centre, Nijmegen, The Netherlands; 13 Department of Obstetrics and Gynaecology, Cancer Center Amsterdam, Amsterdam UMC, Amsterdam, The Netherlands

**Keywords:** Uterine fibroids, Shared decision making, Implementation, User-centered design

## Abstract

**Background:**

Fibroids are non-cancerous uterine growths that can cause symptoms impacting quality of life. The breadth of treatment options allows for patient-centered preference. While conversation aids are known to facilitate shared decision making, the implementation of these aids for uterine fibroids treatments is limited. We aimed to develop two end-user-acceptable uterine fibroids conversation aids for an implementation project. Our second aim was to outline the adaptations that were made to the conversation aids as implementation occurred.

**Methods:**

We used a multi-phase user-centered participatory approach to develop a text-based and picture-enhanced conversation aid for uterine fibroids. We conducted a focus group with project stakeholders and user-testing interviews with eligible individuals with symptomatic uterine fibroids. We analyzed the results of the user-testing interviews using Morville’s Honeycomb framework. Spanish translations of the conversation aids occurred in parallel with the English iterations. We documented the continuous adaptations of the conversation aids that occurred during the project using an expanded framework for reporting adaptations and modifications to evidence-based interventions (FRAME).

**Results:**

The first iteration of the conversation aids was developed in December 2018. Focus group participants (*n* = 6) appreciated the brevity of the tools and suggested changes to the bar graphs and illustrations used in the picture-enhanced version. User-testing with interview participants (*n* = 9) found that both conversation aids were satisfactory, with minor changes suggested. However, during implementation, significant changes were suggested by patients, other stakeholders, and participating clinicians when they reviewed the content. The most significant changes required the addition or deletion of information about treatment options as newer research was published or as novel interventions were introduced into clinical practice.

**Conclusions:**

This multi-year project revealed the necessity of continuously adapting the uterine fibroids conversation aids so they remain acceptable in an implementation and sustainability context. Therefore, it is important to seek regular user feedback and plan for the need to undertake updates and revisions to conversation aids if they are going to be acceptable for clinical use.

**Supplementary Information:**

The online version contains supplementary material available at 10.1186/s12911-024-02637-6.

## Introduction

Fibroids are common non-cancerous uterine growths that can cause heavy and/or prolonged bleeding and symptoms of bulk, such as urinary infrequency, pelvic pressure, and constipation [1]. There are multiple treatment options available for symptomatic uterine fibroids, each one with different advantages or risks. No single treatment is most effective for all patients and not all treatment options are appropriate for all patients, meaning a treatment decision should ideally depend on a patient’s informed preferences and values [2–4].

Conversation aids, like Option Grid™, are brief evidence-based interventions designed to support this process, called shared decision making [5–7]. Our definition of a conversation aid is a patient decision aid that is used to facilitate a shared decision making conversation between a patient and a health professional [5, 8]. They are designed for encounter-based use but can be tailored to be used before and after an encounter [7]. Option Grids are written in plain language with high readability (6th grade or 12 years old). They are developed using an iterative user-centric process compatible with the International Patient Decision Aids Standards (IPDAS) [9]. A previous version of the uterine fibroids Option Grid has been shown to facilitate shared decision making in the context of uterine fibroids treatment decisions [10, 11].

Conversation aids with pictures are shown to be especially useful in increasing understanding and information recall [12–16]. According to a recent systematic review, pictures in health information largely improve comprehension for lower health literacy populations [16]. For displaying risk information, prior studies have revealed varying format preferences and levels of understanding [17, 18].

IPDAS indicates that relevant users of a conversation aid should be embedded in the development of the interventions [19]. A recent systematic review found that conversation aids are frequently developed using user-centered participatory approaches [20]. User-centered design approaches may improve the implementation of conversation aids [21]. User-centered participatory approaches are often iterative and require multiple steps [20]. We know of no existing text-based or picture-enhanced uterine fibroids conversation aids being widely implemented in diverse clinical settings. In a multi-site implementation project of two uterine fibroids conversation aids, our broad project team (including researchers, clinicians, and patients with experience with symptomatic uterine fibroids) developed and iterated two conversation aids for uterine fibroids [22]. Our first objective for this research was to develop a current and acceptable text-based Option Grid and a picture-based version and provide translated versions in Spanish using a multi-phase user-centered participatory approach [22, 23]. Our second objective was to outline the iterative adaptation approach that keeps the Option Grids current and relevant using FRAME, an expanded framework for reporting adaptations and modifications to evidence-based interventions [24].

## Methods

All methods and results are in alignment with the COnsolidated criteria for REporting Qualitative research (CORE-Q) checklist criteria [25]. Please see supplemental file 1.

Option Grid patient conversation aids are created by synthesizing evidence-based information about the benefits and harms of available treatment and screening options, and communicating those findings in a patient-friendly tabular format. We followed a multi-phase user-centered participatory approach to developing and adapting the uterine fibroid treatment options conversation aids. Multiple steps are common for user-centered participatory approaches to conversation aid development [20]. Specifically, there were six documented phases of this development work.

1. Create initial textual content for uterine fibroids treatment options Option Grid
2. Create prototypes of the Option Grid (text-based) and Picture Option Grid (picture-enhanced) and seek feedback from stakeholders
3. Conduct a focus group with the broader project’s Community Advisory Board
4. Conduct user-testing interviews with end-users of the Option Grid
5. Translate and test for acceptability the final approved Option Grid and Picture Option Grid
6. Track adaptations made to the Option Grid and Picture Option Grid

### Phase 1—Initial development of content

#### Setting and participants

EBSCO DynaMed, a group of “clinical experts, scientists, methodologists, specialized medical writers and editors and medical librarians,” generated the initial content of the uterine fibroids Option Grid [26].

#### Procedure

EBSCO DynaMed’s editorial process includes the following steps: (1) Identifying the evidence, (2) Selecting the best evidence available, (3) Critical appraisal, (4) Objectively reporting the evidence, (5) Synthesizing multiple evidence reports, (6) Basing conclusions on the evidence, and (7) Updating daily [27]. Please see supplemental file 2 for an abbreviated description of the development process EBSCO DynaMed uses to develop Option Grids.

Prior to the start of this study, EBSCO DynaMed followed a 45-step process to create the initial content for the uterine fibroids treatment Option Grid (see supplemental file 2). The process involved topic scoping frequently asked questions scoping, evidence gathering, evidence evaluation and synthesis, patient-friendly translation (6th-grade reading level and below), and finalization review.

### Phase 2—Prototype development

#### Setting and participants

This study is part of a broader randomized stepped-wedge implementation study of the uterine fibroids Option Grids [22]. The five participating sites that were randomized to implement the Option Grids at different time points are gynecology clinics at (1) Dartmouth-Hitchcock Medical Center in Lebanon, New Hampshire; (2) Washington University/Barnes-Jewish Hospital in St. Louis, Missouri; (3) Montefiore Medical Center in Bronx, New York; (4) Brigham and Women’s Hospital in Boston, Massachusetts; and (5) Mayo Clinic in Rochester, Minnesota. Sites were chosen based on their diversity (racial, ethnic, and geographic) and interest in and capability of implementing shared decision making tools.

The iterative prototype development process included our broader project’s Community Advisory Board (CAB) and Steering Committee members. Our CAB was composed of 10 individuals. Among five patient partners, two represented different national fibroid advocacy organizations, and two were Black women. Patient partners ranged in geographical location, including rural and urban. Other CAB members included one payer organization representative, one national health insurance clinician representative, and one expert clinician in fibroid care. We selected members of the CAB for their experience or expertise in the multifaceted subject matter of uterine fibroids. They participated in quarterly meetings during the full project duration (June 2018 to January 2023) and were included in regular project communications and requests for feedback. The broader project’s Steering Committee was composed of seven clinician stakeholders, all patient partners, participating site research staff, and two subject matter expertise stakeholders. They participated in quarterly meetings during the full project duration and were included in regular project communications and requests for feedback.

#### Procedure

Working with a professional illustrator and based on synthesized and summarized content from EBSCO DynaMed**,** researchers (M-AD and DS), in collaboration with other project members, created prototypes of Option Grid (text-based and in a tabular format) and Picture Option Grid (picture-enhanced) and then sought feedback from numerous stakeholders, including individuals with symptomatic uterine fibroids. We received feedback at our first site visits to each of the project sites in January and February 2019, and we also received feedback at regularly scheduled monthly team meetings, Steering Committee meetings, and CAB meetings during 2019. We collated Phase 2 feedback in a spreadsheet the project team used to relay information to EBSCO and the project’s illustrator.

### Phase 3—Feedback for picture-enhanced conversation aid

#### Setting and participants

Focus group participants included the project’s CAB. The focus group was conducted using Dartmouth’s HIPAA-compliant Zoom.

#### Procedure

A researcher (DS) conducted a focus group with the CAB in 2019 using an interview guide that explored their overall thoughts about the recently updated Picture Option Grid. The interview guide was also used to seek their opinions about the layout, organization, colors, pictures, and bar graphs in the Picture Option Grid. The interview guide was reviewed and finalized by the Dartmouth project team before the focus group (see supplemental file 3).

Focus group participants had time to review the Picture Option Grid in advance. Planned discussion topics included overall thoughts and opinions about the layout, organization, and colors; specific questions about the pictures and illustrations and the bar graph representations of the statistical information; and specific areas that could be improved. The focus group was audio recorded and transcribed verbatim (DS).

##### Interviewer characteristics

The researcher who conducted the focus group with the project’s CAB as well as the user-testing interviews with individuals with symptomatic uterine fibroids (DS) was a female research assistant who held a bachelor’s degree. The researcher had previous experience conducting focus groups and was trained in qualitative interview methods such as probing and redirection. The researcher had an established relationship with the project’s CAB since she facilitated all quarterly CAB meetings. However, she did not have an established relationship with the user-testing interviewees. Still, they were made aware of her involvement in the broader project and knew of the broader project’s reasons for conducting the study.

### Phase 4—User-testing versions of both conversation aids

#### Setting and participants

We identified a convenience sample of individuals with symptomatic uterine fibroids for the user-testing interviews. We worked with a CAB member to post a brief summary about participation in the study on their advocacy organization's Facebook page, which includes uterine fibroids patients across the United States. Interested individuals reached out by email to participate.

Seventeen individuals were approached by research staff (DS) via email to participate in the user-testing interview. Five individuals did not respond to the email, and three individuals could not participate due to the timing and technological requirements to conduct the interview. Nine total interviews were conducted with patients with symptomatic uterine fibroids. No other individuals were present during participant interviews.

#### Procedure

**Fig. 1:**
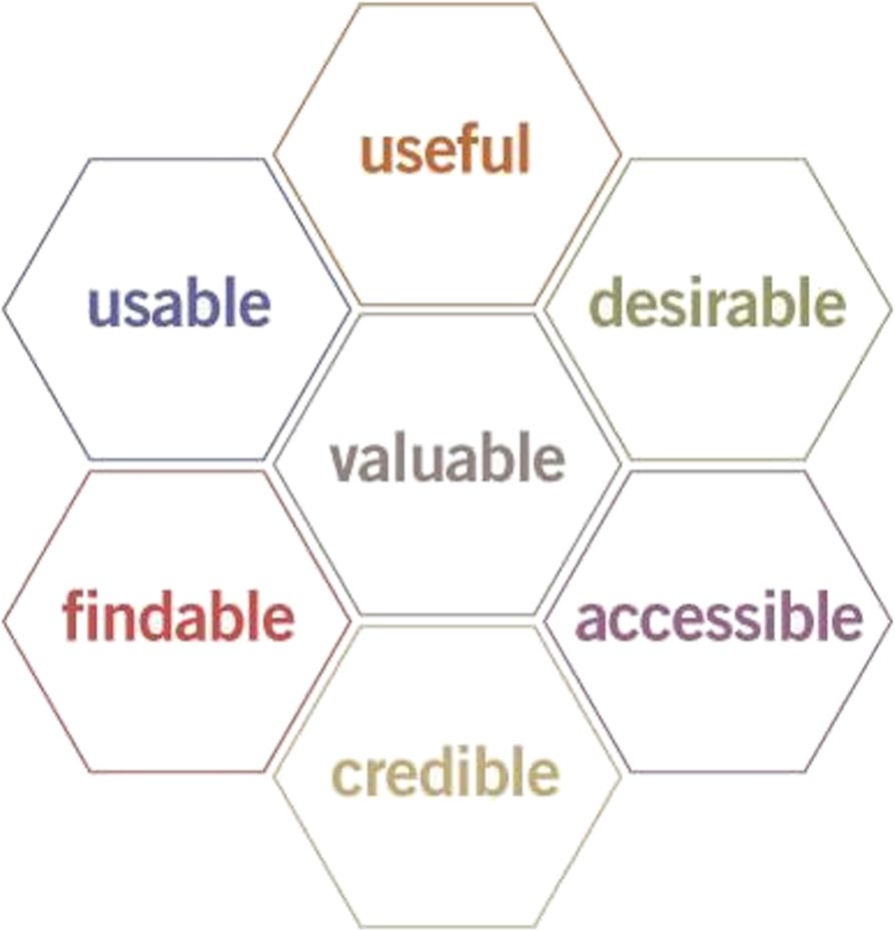
Morville’s user-experience ‘honeycomb’ framework

We developed a user-testing interview guide and analyzed interview transcripts using Morville’s user experience framework [28]. This framework has previously been used to user-test conversation aids.[[29–31] The framework uses seven domains associated with a user’s experience of an intervention: usability, usefulness, credibility, desirability, value, findability and accessibility (see Fig. 1) [32].

The first domain, usable, specifies that the product delivered (the conversation aid), needs to be simple and easy to use. The second domain, useful, says that the product needs to fill a need. Desirable, the third domain, requires that the visual aesthetics of the product need to be attractive, easy to translate, minimal, and to the point. The fourth domain, findable, necessitates that the information provided needs to be easy to navigate. The navigational structure should be set up in a way that makes sense. Accessible, the fifth domain, notes that the product should be designed so that all kinds of users (at all levels of ability) can have the same experience. The second to last domain, credible, describes that the company that makes the product and the product itself needs to be trustworthy. The final domain, valuable, is a compilation of all the other domains. A product cannot be deemed valuable without satisfying all seven domains.

The Phase 4 user-testing interview guide was reviewed by the Dartmouth project team before commencing user-testing interviews (see supplemental file 4). The semi-structured interviews were conducted with a convenience sample of nine individuals over Zoom teleconferencing system in July 2019. The researcher (DS) conducted the interviews to assess the usability, acceptability, and accessibility of the final versions of the two conversation aids. The researcher (DS) contacted individuals and conducted interviews until data saturation was reached. Each user-testing participant was compensated $15 for their interview. The interviews were recorded and transcribed using a HIPAA-compliant transcription company. All recordings and transcriptions were stored using Dartmouth Sharepoint, a HIPAA-compliant web platform. As the transcriptions were verbatim, the researcher (DS) did not take field notes while conducting the interviews.

### Phase 5—Translation of conversation aid content

#### Setting and participants

A bilingual researcher assisted in the Translation, Review, Adjudication, Pretest, Documentation (TRAPD)-adapted translation procedure. The same bilingual researcher identified a convenience sample of native Spanish speakers living in the United States using their social network.

#### Procedure

Our TRAPD-adapted translation approach included the four main stages: (1) Two Spanish speakers created independent translations of the original text; (2) a bilingual reviewer compared the original text, translation 1, and translation 2 and either selected the preferred translation or produced a third translation that built on the previous two; (3) the bilingual reviewer met with the project team to review and reconcile translations by consensus; and (4) the resulting translation was tested via cognitive debriefing interviews [33]. A bilingual researcher conducted the semi-structured cognitive debriefing interviews with a convenience sample of seven native Spanish speakers residing in the United States to ensure the understanding and readability of the uterine fibroids conversation aids. Each cognitive interview participant received a $10 honorarium.

### Phase 6—Tracking continuous adaptations

After gathering all of the above data, we reflected and adapted FRAME to describe the adaptations to the Option Grids throughout the course of the implementation project.

### Analysis

Two researchers (DS and RF) independently coded the Phase 3 focus group and Phase 4 user-testing interviews. A third researcher (MAD) was available for arbitration. The Phase 3 focus group was inductively coded to identify elements that pertain to the usability, acceptability, and accessibility of the Picture Option Grid. The Phase 4 user-testing interviews were analyzed in two rounds. One researcher (DS) conducted the first round of coding solely looking for explicit suggested changes on the tools so the broader project team could finalize the two tools. Two independent researchers (DS and RF) simultaneously conducted the second coding round using framework analysis (based on the ‘honeycomb’ framework). The transcripts were inductively coded to identify elements that pertained to the usability, acceptability, and accessibility of both of the tools. We used ATLAS.ti software for our coding and codebook development. All codes were reviewed by the three researchers (DS, RF, MAD—expert arbitrator) to determine the final major and minor themes.

## Results

### Phase 1 EBSCO content creation

The first Option Grid developed by EBSCO in December 2018 listed six treatment options for uterine fibroids in no particular order or arrangement but separated into two separate grids because of space issues. The first grid listed information for treatment with hormones, medicine without hormones, and myomectomy. The second grid listed information for fibroid destruction, endometrial ablation, and hysterectomy.

### Phase 2 prototype development results

Based on stakeholder feedback of the original version that EBSCO created, there was a push to be more specific about the names of the treatment options/procedures, add “*watch and wait*” as an option, organize the options by treatment intensity, and add brief definitions for some of the treatment options in the top row. Two more versions were developed after collecting feedback (March 2019, April 2019).

At this point, we had worked with the illustrator on developing a picture-enhanced prototype of the Option Grid (called Picture Option Grid). The illustrations were originally drafted in March 2019, and a second round of illustrations was commented on in April 2019. The first prototype of the Picture Option Grid was completed in May 2019 in preparation for the Phase 3 focus group.

### Phase 3 focus group results

Our focus group was held at a regularly scheduled CAB quarterly meeting in May 2019. Six out of 10 members (3 patient partners, 1 clinician, 2 researchers) were present. The focus group lasted thirty minutes and covered questions specific to the Picture Option Grid, as we were in the illustration phase of the intervention development at that time.

The focus group participants had a positive appraisal of and did not suggest any major changes to the Picture Option Grid. They emphasized the importance of the concise nature of the conversation aid. Specific feedback was collected on certain illustrations, including clarifying the meaning of the traffic lights representation and making the bar graphs easier to understand. However, some focus group participants agreed that the bar charts might be difficult for some to interpret.

*“I like the traffic light symbol. I think it’s clear. I think the improved graphics are much easier to read and comprehend.”*

*“I think you could clean them (traffic lights) up a little bit. I’m not sure the people walking in the middle are necessary or the line through the yellow and red is necessary.”*

*“I usually see bar graphs vertically. The vertical bar charts are clearer. I also don’t love the icons, but I think it’s going to have to do with how people best receive and interpret information.”*

The patient partners also focused on the location and size of the fibroids illustrations throughout the Picture Option Grid, and we adjusted our illustrations accordingly. Participants agreed that the Picture Option Grid is a “*nice, clean depiction that we are being mindful of using excess words or images.*”

### Phase 4 user-testing results

Participants (*n* = 9) were all women who had experience with symptomatic uterine fibroids and ranged in age from 32 to 50 (an average of 42 years old). All interview participants had higher education: one associate’s degree, four master’s degrees, and four doctorates. Interview participants ranged in location from California to New York. About half of the participants interviewed identified as Black. Interviews were between 15 min and 1 h long. See Table 1 for user-testing interview participant characteristics.

**Table 1:** User-testing interview participant demographics

**Age**	Average	42
	Range	32–50
**Education**	Associate’s	1
	Master’s	4
	PhD	4
**Race/ethnicity**	Black	4
	White	2
	Missing data	3
**Gender**	Female	9
**Menopause stage**	Premenopause	5
	Perimenopause	1
	Missing data	3

Feedback collected during the interviews included comments about the image of the needle for uterine artery embolization being too intimidating and that it should be reduced in size. Comments were also made about the graph fill needing to be a different color to provide more contrast. Lastly, two interview participants mentioned it would be convenient to have the conversation aids available online.

The results below are organized by each domain of Morville’s user experience honeycomb framework.

#### Usable

Overall, the interview participants found that the conversation aids help to compare options and are easy to understand. Participants who were interviewed felt that the two conversation aids helped compare the numerous treatment options for symptomatic uterine fibroids. They reported the amount of information provided in the conversation aids was sufficient to help facilitate a shared decision making discussion between the patient and clinician.

“*I think that *[the conversation aid] *is a great way to make a decision or at least have help with the decision because it’s a lot of information to take in. Not everybody is a surgery person, not every case is exactly the same. To have choices really makes the difference for people.*” - ID 01

Some participants found the text-only Option Grid easier to understand because of its simpler layout. Most participants conceptually liked the idea of the Picture Option Grid and could understand how the pictures would be helpful for comprehension. Some participants had specific feedback regarding certain pictures and their meanings, including the bar graphs. Some found the bar graphs redundant and found that looking at the accompanying text was more helpful.

“*I would say this one [Picture Option Grid] probably lends itself to increasing the potential of understanding and comprehension because you can envision your body when you look at this, when you absorb the information. The first one [Option Grid] appeals to the analytical mind, such as myself, particularly when you’ve had roles and responsibilities using that type of data. From an intellectual perspective, the first one is great because it still gives you what you need. From the emotional response and reaction, the graphics do a better job in terms of the consultation with the doctor.*” - ID 02

#### Useful

Overall, the interview participants thought the tools had good questions, good information, and served as a good starting point.

The interview participants felt that using the tools was practical, given the flexibility of use, and would help start the conversation about their treatment options. A couple of patients thought that the statistics provided in both conversation aids were not helpful.

“*I think it’s a great conversation starter. I think that the whole idea here is if we’re talking upfront, it does a good job of opening the dialogue to the deeper conversations of practical application, then of selection of choice.*” - ID 04

*“...when I was discussing it with my doctor, I didn’t have any of these and I would try to think of questions to ask. I would almost always come out with the question after I left the appointment. If this was there, then I could only have it there and then she could expound on the answers that were already given to help make an informed decision.”* - ID 02

“*The percentages are really useless to me.*” - ID 08

Participants thought the idea of receiving the conversation aids before their treatment option discussion would be helpful. They noted it would assist in absorbing the information so they can come to their clinic appointment with more personal, focused, and specific questions. Participants also highlighted the importance of taking the tools home for further deliberation.

“*Yes, because sometimes the doctor is talking and you’re kind of listening but if you have something you can take home and sit down in your own time and go through. Personally, for me, that works better for me.*” - ID 01

“*I think it would be practical to receive it before the appointment.*” - ID 04

“...*I think having it beforehand might lessen some of that feeling because you’re processing it, you’re thinking about it, and then when you get to the doctor and you want to sit down and talk, he or she can clarify things for you.*” - ID 01

#### Desirable

Overall, participants preferred the Option Grid for its more concise nature but also enjoyed the picture representations in the Picture Option Grid.

Participants found the Option Grid layout appealing and concise. Among our sample of interview participants, the Option Grid was preferred over the Picture Option Grid. Most participants liked the picture representations in the Picture Option Grid, but they had mixed feelings about the bar graphs.

“*Personally, my preference is just to have the text.*” - ID 02

“*Like I said before, I think the visual is extra helpful to know. (...) I think having a visual representation is useful for all kinds.*” - ID 06

#### Findable

Overall, the participants thought the conversation aids were well-organized. Participants liked the layout of the Option Grid.

Participants found the grouping of the treatment options made sense, and the conversation aids were well-organized. They also felt the Option Grid was easier to navigate because of its tabular structure.

“*I think that it’s [Option Grid] nice. It’s like a crosswalk type of approach, so I think it’s a familiar format for people.*” - ID 08

“*The grouping of treatment options makes sense considering they represent different levels of severity of options.*” - ID 09

“*I like the way that it breaks out into expectations, to what’s actually involved – the expectations, I like the discussion of symptoms and I like kind of understanding the spectrum of risk*.” - ID 08

#### Accessible

Participants felt the conversation aids were broadly accessible because they used plain language. However, the bar graphs may be difficult for some people to understand. Participants have different preferences for which conversation aid they would want to receive. They also highlighted the importance of providing conversation aids in other languages or ensuring an interpreter is available.

“*I think the text is easy to read. I don’t feel like you have to have any kind of medical or scientific background to understand what the text is saying.*” - ID 08

“*You have...the bars - I don’t know if that’s necessarily helpful for your average woman out there or average education level.*” - ID 02

“*I did like the first one a little bit better [Option Grid], but this one [Picture Option Grid], I do think it’s good if you’re a visual learner. I guess it depends on your style of what you prefer.*” - ID 05

#### Credible

Participants trusted the information in the conversation aids, provided that it was coming from their doctor. Nobody interviewed mentioned that conflict of interest information would be helpful or necessary in the conversation aid.

“*I’ve done some research on some of the ways to treat fibroids, and it looks very similar, so it’s definitely the treatments that have been out there.*” - ID 05

#### Valuable

Both tools were deemed valuable by the participants. Participants found both pros and cons for each conversation aid, highlighting the importance of individual preference in choosing which conversation aid to use.

“*I think it’s very helpful. Like I said before, I would like to have had something like this when I first was told that I have fibroids.*” - ID 03

“*For me, it’s a personal choice because everybody is different in their learning methods.*” - ID 02

### Phase 6 FRAME results

As the broader implementation project went on, we continued to collect feedback from clinical and patient stakeholders. We made subsequent modifications to both the text-based Option Grid and the picture-enhanced Picture Option Grid. Please see Table 2 below which describes the major changes to the tools over time according to FRAME. All adaptations were (1) at the intervention level of modification delivery, (2) content level modifications, and (3) considered fidelity consistent in that it kept the tools relevant and useful. Any adaptations to the tools over time resulted in updated Spanish translations as well.

**Table 2:** FRAME results for main changes made to the conversation aids

**What is modified?**	**When/How**	**Proactive/Reactive**	**Who determined?**	**Reasons for modification**
Adding "Watch and Wait" as an option	Before implementation	Reactive	Patient and stakeholder partners	Patients described they want an option that isn't a medical intervention
Organizing options by medicine treatments vs. procedural treatments	Before implementation	Reactive	Patient and stakeholder partners, site principal investigators	Patients, stakeholder partners, and site principal investigators brainstormed a way to better present the treatment options that make sense when having the shared decision making conversation. By organizing for medicine vs. procedural options, it helps the providers and patients narrow the conversation
Adding information about time to return to work	Before implementation	Reactive	Patient and stakeholder partners	Patient partners stated that this is an important question to answer for women who are still working (most pre- and peri-menopausal women)
Adding radiofrequency ablation as treatment option—taking out endometrial ablation as treatment option	During implementation	Reactive	Participating UPFRONT clinicians	Suggested to add by participating UPFRONT clinicians as it was an emerging treatment that became a more common practice and treatment option across our sites
Adding "emerging treatments" as treatment option	During implementation	Reactive	Participating UPFRONT clinicians	Allows for more conversation between the patient and clinician regarding what other options are available that are not listed
Enlarging the illustrations in the Picture Option Grid	During sustainability	Reactive	Participating UPFRONT clinicians	Suggested to add by participating UPFRONT clinicians so that illustrations are focal point in Picture Option Grid

**Fig. 2:**
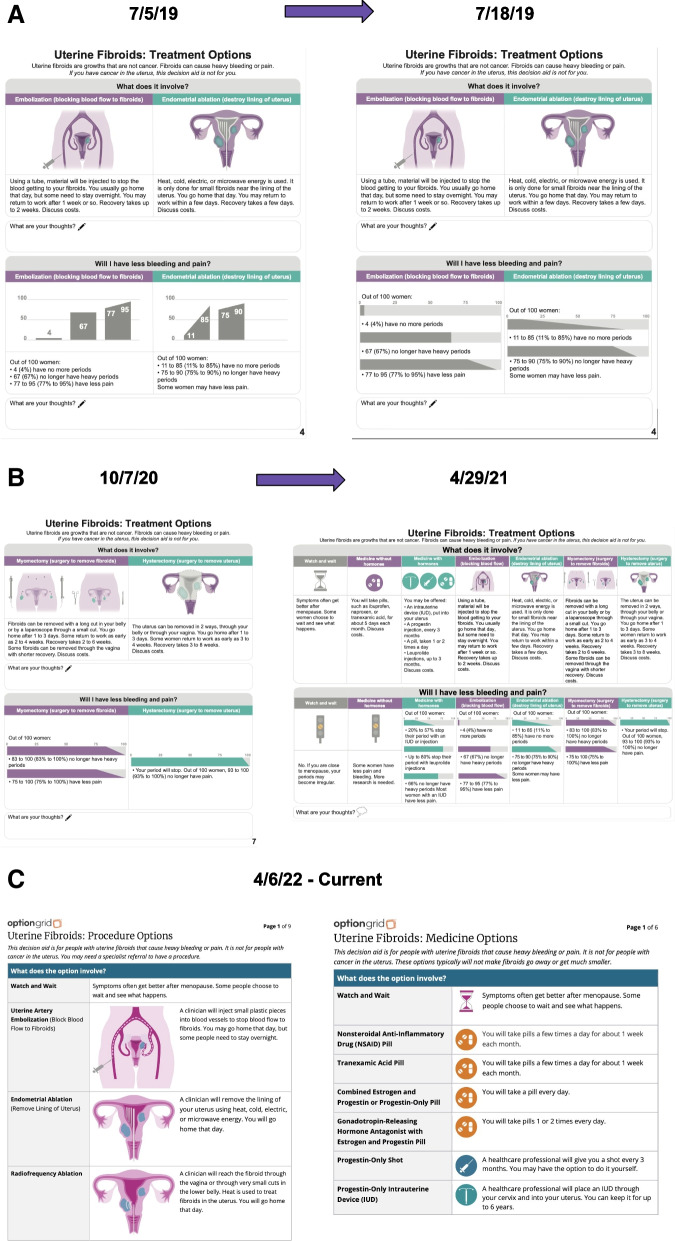
**a**-**c** Evolution of the picture option grid

Please see Fig. 2a-c for a visualization of the modifications to the Picture Option Grid over the course of the project. See supplemental files 5–8 to view full versions of the Picture Option Grid shown in Fig. 2a-c.

## Discussion

The six phases of developing and tracking adaptations of the conversation aid informed us that continuous updating of the Option Grid is needed for successful implementation and sustainability, especially if the tools will be used across diverse settings over many years. Phase 3 focus group members suggested minor changes to the bar graphs and traffic lights on the Picture Option Grid. Overall, they liked the brevity of the updated Picture Option Grid and did not suggest any major changes. Phase 4 user testing interview participants found that both conversation aids met the criteria for satisfactory user experience. In Phase 6, we organized the major adaptations to the tools using FRAME, noting most main changes were reactive and determined by patient/stakeholder partners and participating clinicians.

A consistent user-centered participatory approach that collects and responds to user and stakeholder feedback facilitates continuous adaptation of the conversation aids. While doing this can slow down the actual implementation work, it is necessary to ensure the usability and acceptability of the conversation aids for sustainability.

### Comparison with other literature

We observed that both patients and clinicians had varying preferences for one format over the other (Option Grid versus Picture Option Grid). While we accept that preferences will determine utilization, we emphasized the evidence showing that pictures and visual risk communication strategies can help increase knowledge and understanding, especially for people with lower health literacy [16]. In our user-testing sample, bar graphs were moderately understood with preference for the text accompanying them, also seen in another study [34]. Research also shows that bar graphs may be more difficult to understand for individuals with lower graph literacy and fewer years of education [35].

Another major finding from the user-testing interviews was the benefit to patients of receiving the conversation aids before their visits with their clinician. Bunzli’s study on an orthopedic surgery decision aid also reported this [36]. Being able to read and process the information ahead of visits helps patients better prepare and take part in discussions about their treatment options.

Our finding that there is a need to continually adapt patient decision aids during implementation processes is echoed by others [37]. Holtrop et al. emphasize the importance of mental models in implementation science and that adaptations are, therefore, a consequence of learning by understanding the perspectives of end-users [38]. Adaptations should be appreciated, documented, and evaluated [39]. Additionally, the iterative process of responsive continuous adaptation created a “ripple effect”: end-users realized that their opinions led to changes and became more interested in successfully implementing the conversation aids [40, 41].

### Strengths and limitations

One strength of this study was the involvement of key stakeholders from the conversation aids’ inception to real-life implementation. This approach required continual interactions on a regular basis, which provided full transparency of the development progress. Our iterative approach also emphasized the importance of adapting the conversation aids to target population needs, which in turn made them more suitable for sustainability.

“*I feel like I have helped to implement and shape this thing, and to kind of put my hat in the ring on behalf of my own personal community, but then the other communities that I know are oftentimes unrepresented at the upfront stages when things are being discussed.*” - Phase 4 user-testing interview, ID 04

In hindsight, we unintentionally overlooked user-testing the final versions of the conversation aids with clinicians, as they are also end-users of the conversation aids in practice. Including all end-users may be the most effective approach to reducing some implementation barriers [42]. Another limitation is that most of our user-testing interview participants were more educated and of higher socioeconomic status, which is not representative of the population with symptomatic uterine fibroids and reduces generalizability.

### Lessons learned and recommendations

In doing our best to follow an iterative user-centered participatory approach, the adaptation and development of the conversation aids took time, effort, and resources. Beyond strictly developing the conversation aids, we also spent over a year using a trial and error approach to how the conversation aids would be printed for real-life use in practice (text-based Option Grid printed as a tear-off pad, Picture Option Grid printed as an individual document). We learned that in future development efforts, it is important to allow enough time for the development and dissemination of conversation aids in diverse clinical settings.

Our recommendations for future development of conversation aids is to use a user-centered participatory approach with all end-users from inception to implementation and sustainability, being mindful of the time it takes to do this successfully with better decisions made. We suggest using the Honeycomb framework in developing and analyzing conversation aids as it provides a useful structure for understanding how end-users perceive and engage with them. We highly recommend keeping conversation aids concise and tailoring them to individual patient’s needs, as this was a major theme highlighted by all stakeholders in the project. Lastly, we recommend incorporating flexibility into a project timeline to allow for continuous adaptations of the conversation aids so they remain useful in an implementation and sustainability context.

## Conclusion

Using a user-centered participatory approach to develop a conversation aid from inception to implementation and sustainability takes time and effort. Still, it provides a framework to systematically include all potential end-users and stakeholders. To successfully implement the conversation aids across a broad audience, we learned the importance of providing concise information while offering customization (to both the clinician and patient). Lastly, allowing for continuous adaptations of a conversation aid is important for the usability and acceptability of the tools so they are better sustained in clinical practice.

## Supplementary Information

Supplementary Material 1.

Supplementary Material 2.

Supplementary Material 3.

Supplementary Material 4.

Supplementary Material 5.

Supplementary Material 6.

Supplementary Material 7.

Supplementary Material 8.

## Data Availability

The dataset analyzed during the current study is not publicly available due to the protection of the human subjects involved in the qualitative interviews. A de-identified copy of the dataset is available from the corresponding author upon reasonable request.
