# Supplementary material for: Continuous adaptation of conversation aids for uterine fibroids treatment options in a four-year multi-center implementation project
Source: BMC Med Inform Decis Mak. 2024 Sep 30;24:277. doi: 10.1186/s12911-024-02637-6 (PMC11441251; doi:10.1186/s12911-024-02637-6)
Supplement: Supplementary file 2 — Supplementary Material 2. [file 12911_2024_2637_MOESM2_ESM.docx]

**DynaMed Decisions: Shared Decision-Making Tools Development Process**

**Scoping**

Each decision aid addresses a healthcare decision where a patient can benefit from shared decision-making. The editorial team gathers background information on the selected healthcare decision to identify the most common and appropriate options, as well as the most frequently asked questions (FAQs). Sources include clinical expertise from specialty and general practice clinicians, clinical practice guidelines and research on the options, patient information sources, and when possible, existing research on patient values and preferences. A critical part of scoping is assessing the extent to which the evidence can answer the FAQs and any important nuances to consider from the evidence. This helps inform how the editorial team will approach answering the FAQs.

**Search and Selection**

The editorial team conducts a thorough search for the best available evidence on the topic of the decision aid. They do systematic searches that start by leveraging DynaMed’s systematic literature surveillance but also involve conducting additional systematic literature searches. The editorial team prioritizes systematic reviews (SRs) when possible, and they identify and evaluate primary research articles when necessary (e.g., when SRs are inadequate or there is additional research since the most current well-done SR) or if they provide additional detail that is not captured by the SRs.

**Critical Appraisal**

The editorial team evaluates each study, whether it is a SR or primary research article. This critical appraisal uses standardized approaches for data extraction and quality assessment, with body of evidence assessments using GRADE methods.

**Synthesis and Plain Language Review**

After finishing critical appraisal, the editorial team synthesizes this information to answer each FAQ based on the best available evidence. This synthesis also uses GRADE methods. Each answer also adopts plain language principles to make the answers understandable to the broadest audience. Each decision aid aims for a grade 6 reading level.

**Final Review**

The resulting decision aid undergoes several rounds of review, including review by an independent specialty clinician in the relevant area of medicine, an independent general practice clinician, and a group of potential users to ensure readability and usability by the public. As a final step, a dedicated group with expertise in medicine, evidence, and patient communication principles reviews the full decision aid.
