# Supplementary material for: Continuous adaptation of conversation aids for uterine fibroids treatment options in a four-year multi-center implementation project
Source: BMC Med Inform Decis Mak. 2024 Sep 30;24:277. doi: 10.1186/s12911-024-02637-6 (PMC11441251; doi:10.1186/s12911-024-02637-6)
Supplement: Supplementary file 3 — Supplementary Material 3. [file 12911_2024_2637_MOESM3_ESM.docx]

**Step 1: Consent process**

Thank you for being here today to talk to us about the latest prototype of the uterine fibroids treatment Picture Option Grid. This conversation will last no longer than 30 minutes. This focus group will be recorded and transcribed in order to analyze your comments and feedback. The recordings will be uploaded onto Dartmouth Sharepoint, which is a HIPAA-compliant platform. Any information reported from this focus group will be de-identified. Are you comfortable with the audio recording?

*Based on the answer, turn the recorder on. Check to be sure the recording light is illuminated. Proceed with the interview.*

The recorder is now on. Do you understand the purpose of this interview and consent to take part?

*Ensure they respond with ‘yes’ (or something similar).*

**Step 2: Review the Picture Option Grid**

The tool you are currently viewing on your screen is called a Picture Option Grid. It will be given to and used with women who are making decisions about their treatment for their symptomatic uterine fibroids. We’ve developed this tool using a community based participatory research approach involving feedback opportunities with the core team, broad study team, and the Community Advisory Board. The textual content that you see has undergone numerous feedback opportunities from all project team members. EBSCO Health develops the textual content using the most current evidence available following a rigorous evidence synthesis process. For the purposes of this focus group, we are looking specifically for feedback on the layout, illustrations, and graphs represented in the Picture Option Grid.

Please take a moment to review the current prototype of the uterine fibroids Picture Option Grid.

**Step 3: Picture Option Grid interview questions**

1. **Now that you have had a chance to look at the Picture Option Grid, what do you think about it overall?**
2. **What do you think of the layout, organization, and colors?**
3. **What do you think of the pictures?**

What are your thoughts about how the uterus is shown?

What are your thoughts about how the fibroids are shown?

What pictures, if any, are confusing?

1. **What do you think of the bar graphs?**

What do you think of the bar graphs that show ranges?

What do you think of the icons associated with the bar graphs?

1. **What can be improved?**
