# Supplementary material for: Continuous adaptation of conversation aids for uterine fibroids treatment options in a four-year multi-center implementation project
Source: BMC Med Inform Decis Mak. 2024 Sep 30;24:277. doi: 10.1186/s12911-024-02637-6 (PMC11441251; doi:10.1186/s12911-024-02637-6)
Supplement: Supplementary file 4 — Supplementary Material 4. [file 12911_2024_2637_MOESM4_ESM.docx]

**Uterine fibroids OG/POG user-testing interview guide**

**Step 1: Consent (2 minutes)**

Thank you for joining this interview today. This interview is being conducted by a research team member from Dartmouth College at The Dartmouth Institute for Health Policy and Clinical Practice. This interview serves to test our final versions of the uterine fibroids Option Grid and Picture Option Grid patient decision aids. This conversation will last no longer than 30 minutes. Your participation is voluntary. You may choose to not answer any or all questions.

The information collected from this interview will be maintained confidentially, and any information reported from this interview will be de-identified. Any questions about this interview may be directed to:

Glyn Elwyn

Tenured Professor and Senior Scientist

1 Medical Center Drive (WTRB, Level 5)

Lebanon, NH 03756

[glynelwyn@gmail.com](mailto:glynelwyn@gmail.com)

With your permission, this interview will be audio-recorded and transcribed in order to analyze your comments and feedback. The recordings will be uploaded onto Dartmouth Sharepoint, which is a HIPAA-compliant platform. The audio recordings will be destroyed after the project duration is complete. Are you comfortable with the audio recording?

*Based on the answer, turn the recorder on and the recording on Zoom. Check to be sure the recording lights are illuminated. Proceed with the interview.*

The recorder is now on. Do you have any questions before we get started?

*After questions:* Do you understand the purpose of this interview and agree to take part?

*Ensure they respond with ‘yes’ (or something similar).*

**Step 2: Demographics** **(1 minute)**

Before we begin the interview, we have a few questions for you to help us understand your background a little more.

1. What is the highest degree or level of school you have completed?
2. What is your age?
3. What state do you live in?
4. Have you experienced uterine fibroids?

Thank you very much for answering these questions.

**Step 3: Introduction to the tools (1.5 minutes)**

The tools you will be viewing on your screen are called Option Grid and Picture Option Grid. Option Grid and Picture Option Grid are patient decision aids. These will be given to and used with women who are making decisions about treatment for their symptomatic uterine fibroids. These tools are designed for use in the clinic visit, although they can be used before or after as well. Accordingly, the tools are meant to be short and concise to spark further conversation about the details of each treatment option.

The Option Grid and Picture Option Grid are separated into 3 sections by type of treatment option. The first section compares “watchful waiting”, “medicine with hormones”, and “medicine without hormones”. The second compares “endometrial ablation” and “uterine artery embolization”. The third compares “myomectomy” and “hysterectomy”. There will also be an online version available of the text-based tool where you can select which options you would like to compare.

We’ve developed these tools using a community based participatory research approach involving feedback opportunities with community stakeholders. A company called EBSCO Health develops the information about each option using the most current evidence available. For the purposes of this interview, we are looking for feedback about how difficult or easy the tools are to understand and use.

**Step 4: Option Grid** **(12 minutes)**

Please take a moment to review the current version of the uterine fibroids Option Grid. I will scroll from the top, and please let me know if I need to go slower.

1. **Now that you have had a chance to look at the Option Grid, what do you think about it overall?**
2. **What do you think of the layout and design?**

What do you think of the grid layout as a way to compare treatment options? **(Findable)**

1. **What do you think about the information in the grid?**

What do you think about the amount of information available?

Do you trust the information provided? **(Credible)**

How easy or difficult is it to understand the text?

Are there any sections that are unclear?

**(4) What are your thoughts about women using this tool with their doctor to help them make a treatment decision? (Usable)**

Do you think women will want to use these tools with their doctor? **(Desirable)**

Do you think using this tool is practical? **(Useful)**

Do you think all types of women with fibroids can use this tool? **(Accessible)**

How helpful do you think this tool would be for women making a treatment decision? **(Valuable)**

**(5) Do you have any more thoughts about this tool?**

**Step 5: Picture Option Grid (13 minutes)**

Please take a moment to review the current version of the uterine fibroids Picture Option Grid. I will scroll from the top, and please let me know if I need to go slower.

1. **Now that you have had a chance to look at the Picture Option Grid, what do you think about it overall?**
2. **What do you think of the layout and design?**

What do you think of the grouping of the treatment options? **(Findable)**

What do you think of the colors?

1. **What do you think of the pictures and the bar graphs?**

What are your thoughts about how the uterus and fibroids are shown?

How well do you think the pictures represent the written content?

Did you see anything about the pictures that you felt was confusing?

Can you please walk me through on how you interpret the bar graphs in “Will I have less bleeding and pain” for “Medicine with hormones”?

**(4) What are your thoughts about women using this tool with their doctor to help them make a treatment decision? (Usable)**

Do you think women will want to use these tools with their doctor? **(Desirable)**

Do you think using this tool is practical? **(Useful)**

Do you think all types of women with fibroids can use this tool? **(Accessible)**

How helpful do you think this tool would be for women making a treatment decision? **(Valuable)**

1. **Do you have any more thoughts about this tool?**
