# Supplementary material for: Continuous adaptation of conversation aids for uterine fibroids treatment options in a four-year multi-center implementation project
Source: BMC Med Inform Decis Mak. 2024 Sep 30;24:277. doi: 10.1186/s12911-024-02637-6 (PMC11441251; doi:10.1186/s12911-024-02637-6)
Supplement: Supplementary file 7 — Supplementary Material 7. [file 12911_2024_2637_MOESM7_ESM.pdf]

Uterine Fibroids: Treatment Options

Uterine fibroids are growths that are not cancer. Fibroids can cause heavy bleeding or pain.
If you have cancer in the uterus, this decision aid is not for you.

What does it involve?

Medicine with hormones	Medicine without hormones	Watch and wait
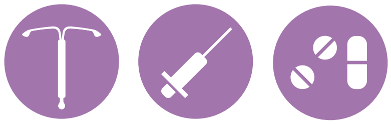	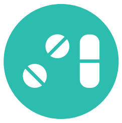	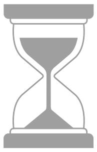
You may be offered:<br>• An intrauterine device (IUD), put into your uterus<br>• A progestin injection, every 3 months<br>• A pill, taken 1 or 2 times a day<br>• Leuprolide injections, up to 3 months<br>Discuss costs.	You will take pills, such as ibuprofen, naproxen, or tranexamic acid, for about 5 days each month. Discuss costs.	Symptoms often get better after menopause. Some women choose to wait and see what happens.

What are your thoughts?

Will I have less bleeding and pain?

Medicine with hormones	Medicine without hormones	Watch and wait
Out of 100 women:<br>0 25 50 75 100<br>• 20% to 57% stop their period with an IUD or injection<br>• Up to 80% stop their period with leuprolide injections<br>• 66% no longer have heavy periods<br>Most women with an IUD have less pain.	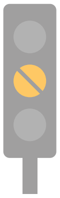<br>Some women have less pain and bleeding. More research is needed.	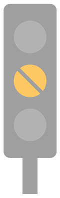<br>No. If you are close to menopause, your periods may become irregular.

What are your thoughts?

Will the fibroids go away or get smaller (in size)?

Medicine with hormones	Medicine without hormones	Watch and wait
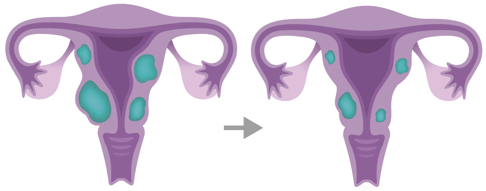	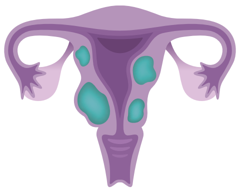	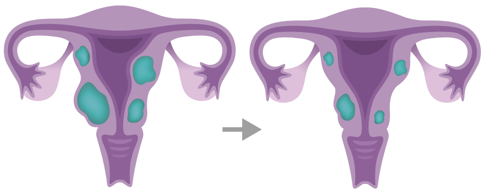
After leuprolide injections, fibroids may get smaller. They grow back when you stop. Other hormone medicines may not help.	No.	Your fibroids may get smaller with menopause and cause less problems. If you are younger, fibroids may get bigger.

What are your thoughts?

Is it safe to get pregnant?

Medicine with hormones	Medicine without hormones	Watch and wait
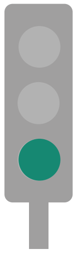	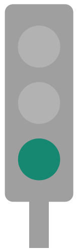	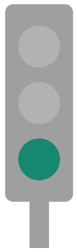
Yes, but you will need to stop the medicine before trying to get pregnant.	Yes.	Yes.

What are your thoughts?

What are the side effects?

Medicine with hormones	Medicine without hormones	Watch and wait
Out of 100 women, up to:	Out of 100 women, up to:	
• 71 (71%) get hot flashes, mainly with leuprolide injections	• 60 (60%) get headaches, mainly with tranexamic acid	• There are no side effects.
• 70 (70%) have irregular bleeding	• 20 (20%) get pain in their stomach	
• 37 (37%) get headaches	• 15 (15%) get nauseous	

What are your thoughts?

What are the more serious risks?

Medicine with hormones	Medicine without hormones	Watch and wait
Out of 100 women with an IUD:		
• 23 (23%) do not get better or have problems with it by 1 year	• Less than 1 out of 100 women (1%) have a stroke, blood clot, stomach bleed, or ulcer.	• There are no serious risks.
• It falls out in up to 10 (10%)		
• Less than 1 (1%) get infection or injury		

What are your thoughts?

Uterine Fibroids: Treatment Options

Uterine fibroids are growths that are not cancer. Fibroids can cause heavy bleeding or pain.
If you have cancer in the uterus, this decision aid is not for you.

What does it involve?

Embolization (blocking blood flow to fibroids)	Endometrial ablation (destroy lining of uterus)
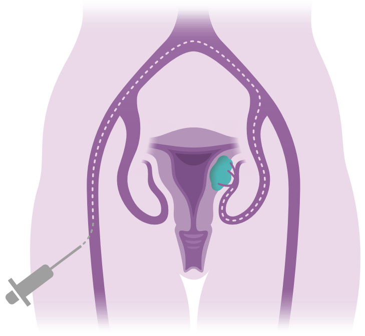	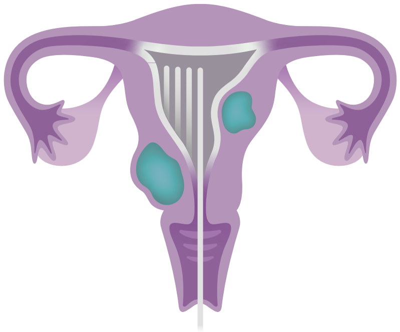
Using a tube, material will be injected to stop the blood getting to your fibroids. You usually go home that day, but some need to stay overnight. You may return to work after 1 week or so. Recovery takes up to 2 weeks. Discuss costs.	Heat, cold, electric, or microwave energy is used. It is only done for small fibroids near the lining of the uterus. You go home that day. You may return to work within a few days. Recovery takes a few days. Discuss costs.

What are your thoughts?

Will I have less bleeding and pain?

Embolization (blocking blood flow to fibroids)

Out of 100 women:

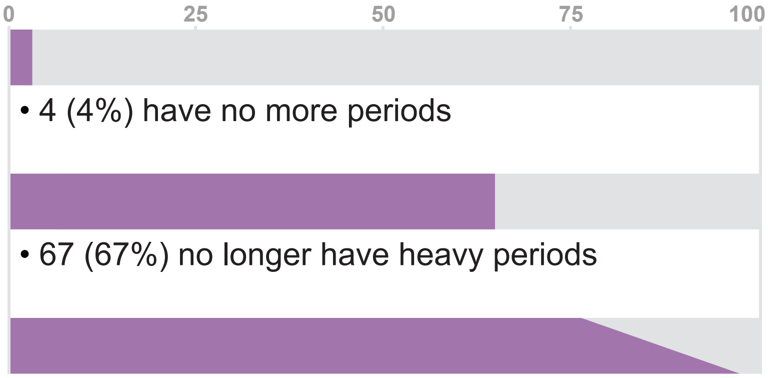

• 77 to 95 (77% to 95%) have less pain

Endometrial ablation (destroy lining of uterus)

Out of 100 women:

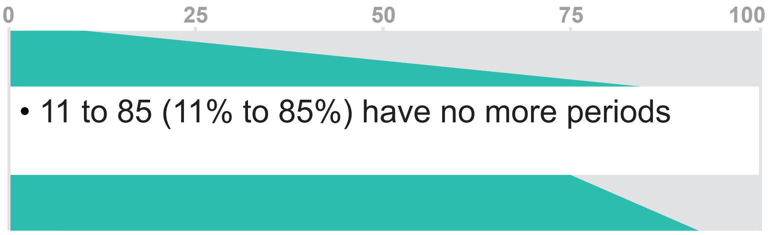

• 75 to 90 (75% to 90%) no longer have heavy periods
Some women may have less pain.

What are your thoughts?

Will the fibroids go away or get smaller (in size)?

Embolization (blocking blood flow to fibroids)	Endometrial ablation (destroy lining of uterus)
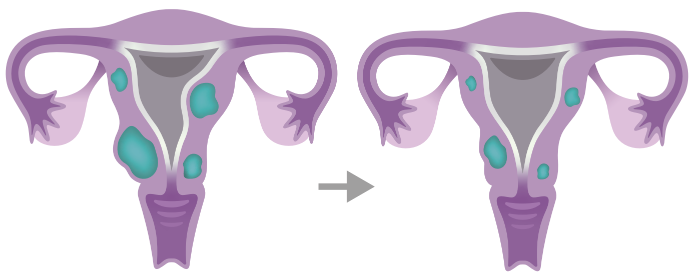	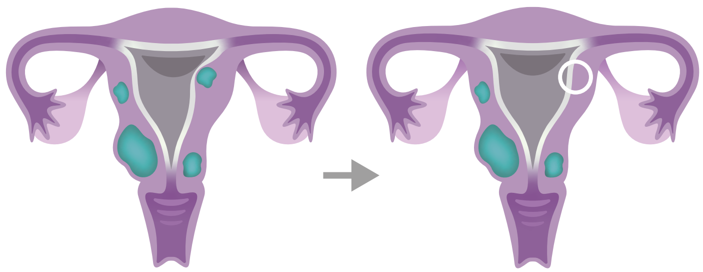
Your fibroids may get smaller.	Research is limited. Very small fibroids near the uterus lining may go away when the uterus lining is removed.

What are your thoughts?

Is it safe to get pregnant?

Embolization (blocking blood flow to fibroids)	Endometrial ablation (destroy lining of uterus)
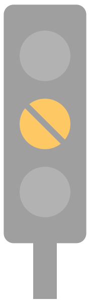	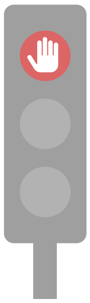
More research is needed to answer this question for this option.	No. There are serious risks to being pregnant. Discuss birth control.

What are your thoughts?

What are the side effects?

Embolization (blocking blood flow to fibroids)

For a short time, out of 100 women, up to:

- 89 (89%) have pain
- 64 (64%) get nauseous
- 21 (21%) have vaginal discharge

Endometrial ablation (destroy lining of uterus)

For a short time, out of 100 women, up to:

- 72 (72%) get cramps
- 36 (36%) get nauseous or throw up due to anesthesia
- 10 (10%) have pain when peeing

What are your thoughts?

What are the more serious risks?

Embolization (blocking blood flow to fibroids)

Out of 100 women:

- 24 (24%) need more surgery by 2 years
- 3 (3%) get an infection
- Less than 1 (1%) get a blood clot

Endometrial ablation (destroy lining of uterus)

Out of 100 women, up to:

- 12 (12%) need more surgery by 2 years
- 6 (6%) get an infection
- 5 (5%) get an injury to their uterus

What are your thoughts?

Uterine Fibroids: Treatment Options

Uterine fibroids are growths that are not cancer. Fibroids can cause heavy bleeding or pain.
If you have cancer in the uterus, this decision aid is not for you.

What does it involve?

Myomectomy (surgery to remove fibroids)	Hysterectomy (surgery to remove uterus)
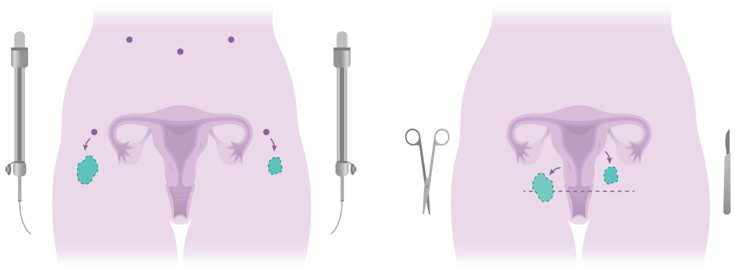	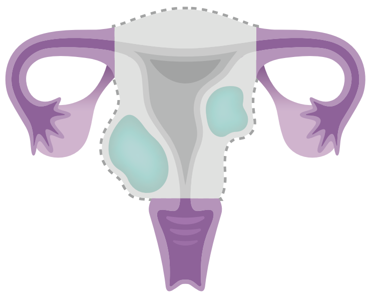
Fibroids can be removed with a long cut in your belly or by a laparoscope through a small cut. You go home after 1 to 3 days. Some return to work as early as 2 to 4 weeks. Recovery takes 2 to 6 weeks. Some fibroids can be removed through the vagina with shorter recovery. Discuss costs.	The uterus can be removed in 2 ways, through your belly or through your vagina. You go home after 1 to 3 days. Some women return to work as early as 3 to 4 weeks. Recovery takes 3 to 8 weeks. Discuss costs.

What are your thoughts?

Will I have less bleeding and pain?

Myomectomy (surgery to remove fibroids)

Out of 100 women:

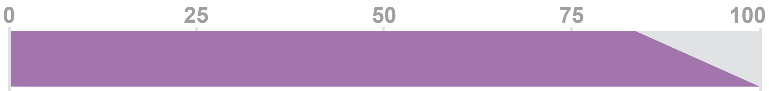

• 83 to 100 (83% to 100%) no longer have heavy periods

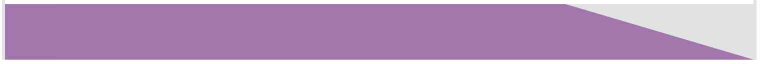

• 75 to 100 (75% to 100%) have less pain

Hysterectomy (surgery to remove uterus)

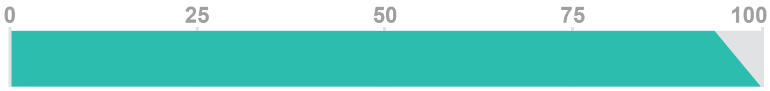

• Your period will stop. Out of 100 women, 93 to 100 (93% to 100%) no longer have pain.

What are your thoughts?

Will the fibroids go away or get smaller (in size)?

Myomectomy (surgery to remove fibroids)	Hysterectomy (surgery to remove uterus)
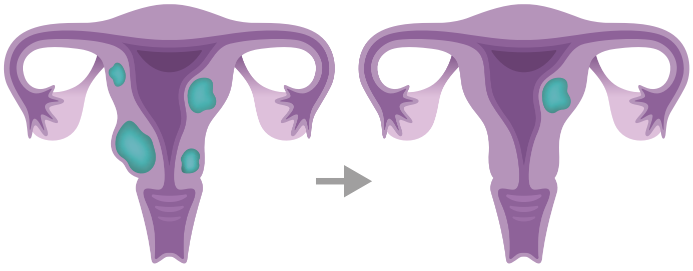	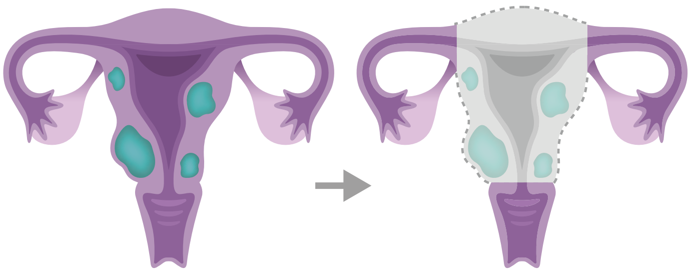
Most fibroids can be removed.	Your fibroids will be removed with your uterus.

What are your thoughts?

Is it safe to get pregnant?

Myomectomy (surgery to remove fibroids)	Hysterectomy (surgery to remove uterus)
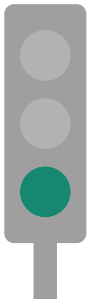	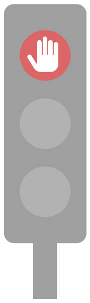
Yes. You may need to deliver by C-section.	No. You will no longer be able to get pregnant.

What are your thoughts?

What are the side effects?

Myomectomy (surgery to remove fibroids)

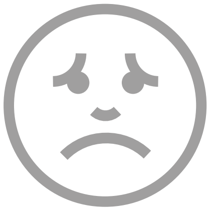

Pain and nausea or throwing up due to anesthesia are common after surgery.

Hysterectomy (surgery to remove uterus)

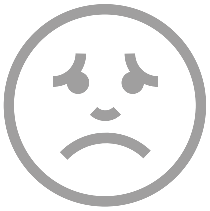

Pain and nausea or throwing up due to anesthesia are common after surgery. If your ovaries are also removed, you will go into menopause.

What are your thoughts?

What are the more serious risks?

Myomectomy (surgery to remove fibroids)

Risks depend on how the myomectomy is done. Out of 100 women, up to:

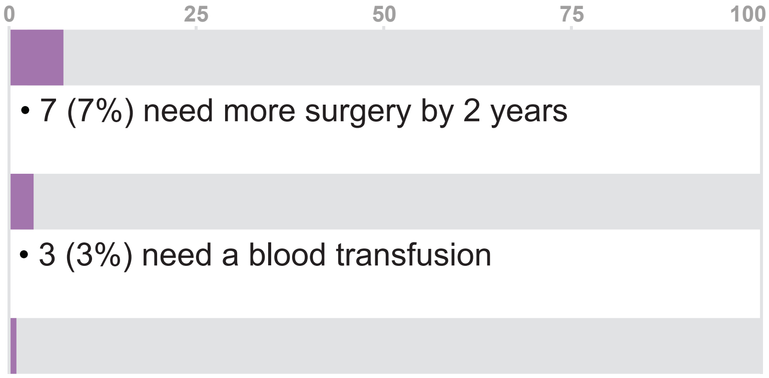

- 7 (7%) need more surgery by 2 years
- 3 (3%) need a blood transfusion
- Less than 1 (1%) need a hysterectomy during surgery

Hysterectomy (surgery to remove uterus)

Risks depend on how the hysterectomy is done. Out of 100 women, up to:

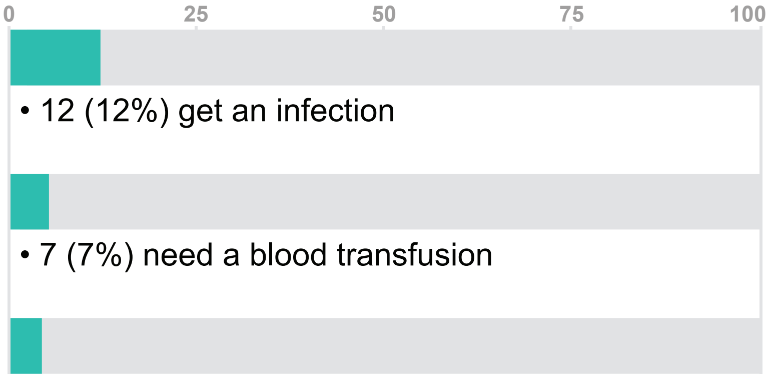

- 12 (12%) get an infection
- 7 (7%) need a blood transfusion
- 6 (6%) get a urinary system injury

What are your thoughts?
