# Supplementary material for: Continuous adaptation of conversation aids for uterine fibroids treatment options in a four-year multi-center implementation project
Source: BMC Med Inform Decis Mak. 2024 Sep 30;24:277. doi: 10.1186/s12911-024-02637-6 (PMC11441251; doi:10.1186/s12911-024-02637-6)
Supplement: Supplementary file 8 — Supplementary Material 8. [file 12911_2024_2637_MOESM8_ESM.pdf]

Uterine Fibroids: Treatment Options

Uterine fibroids are growths that are not cancer. Fibroids can cause heavy bleeding or pain. *If you have cancer in the uterus, this decision aid is not for you.*

What does it involve?

Watch and wait	Medicine without hormones	Medicine with hormones	Embolization (blocking blood flow)	Endometrial ablation (destroy lining of uterus)	Myomectomy (surgery to remove fibroids)	Hysterectomy (surgery to remove uterus)
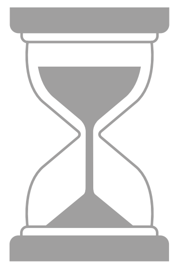	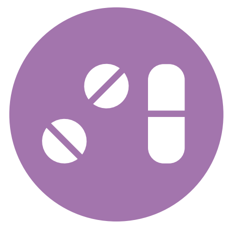	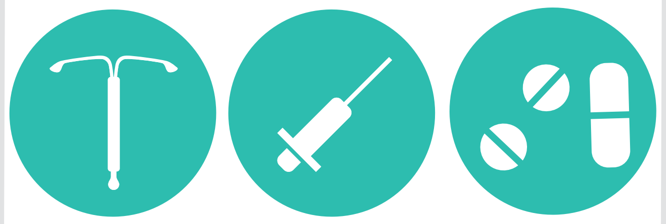	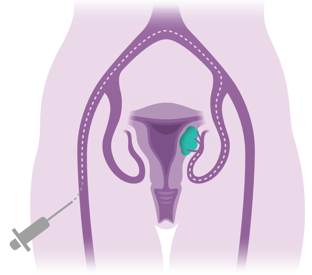	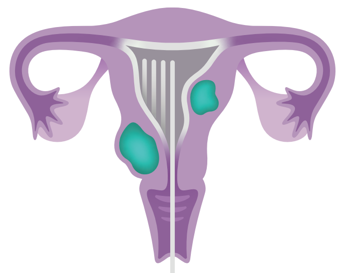	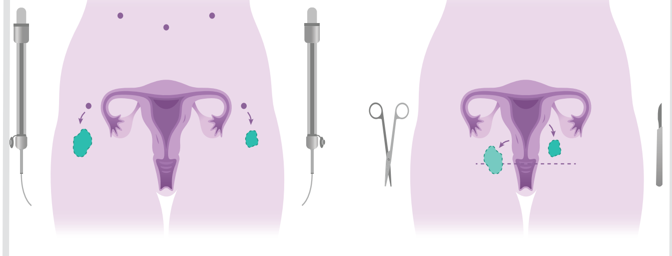	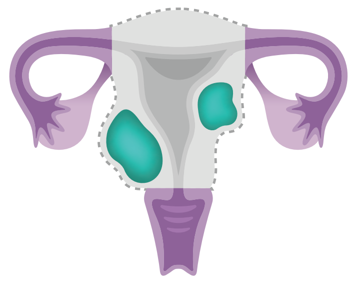
Symptoms often get better after menopause. Some women choose to wait and see what happens.	You will take pills, such as ibuprofen, naproxen, or tranexamic acid, for about 5 days each month. Discuss costs.	You may be offered:<br>• An intrauterine device (IUD), put into your uterus<br>• A progestin injection, every 3 months<br>• A pill, taken 1 or 2 times a day<br>• Leuprolide injections, up to 3 months.<br>Discuss costs.	Using a tube, material will be injected to stop the blood getting to your fibroids. You usually go home that day, but some need to stay overnight. You may return to work after 1 week or so. Recovery takes up to 2 weeks. Discuss costs.	Heat, cold, electric, or microwave energy is used. It is only done for small fibroids near the lining of the uterus. You go home that day. You may return to work within a few days. Recovery takes a few days. Discuss costs.	Fibroids can be removed with a long cut in your belly or by a laparoscope through a small cut. You go home after 1 to 3 days. Some return to work as early as 2 to 4 weeks. Recovery takes 2 to 6 weeks. Some fibroids can be removed through the vagina with shorter recovery. Discuss costs.	The uterus can be removed in 2 ways, through your belly or through your vagina. You go home after 1 to 3 days. Some women return to work as early as 3 to 4 weeks. Recovery takes 3 to 8 weeks. Discuss costs.

Will I have less bleeding and pain?

Watch and wait	Medicine without hormones	Medicine with hormones	Embolization (blocking blood flow)	Endometrial ablation (destroy lining of uterus)	Myomectomy (surgery to remove fibroids)	Hysterectomy (surgery to remove uterus)
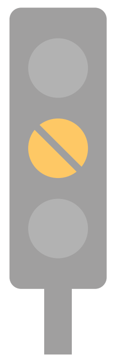	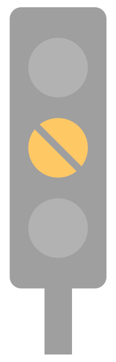	Out of 100 women:<br>• 20% to 57% stop their period with an IUD or injection<br>• Up to 80% stop their period with leuprolide injections<br>• 66% no longer have heavy periods Most women with an IUD have less pain.	Out of 100 women:<br>• 4 (4%) have no more periods<br>• 67 (67%) no longer have heavy periods<br>• 77 to 95 (77% to 95%) have less pain	Out of 100 women:<br>• 11 to 85 (11% to 85%) have no more periods<br>• 75 to 90 (75% to 90%) no longer have heavy periods<br>Some women may have less pain.	Out of 100 women:<br>• 83 to 100 (83% to 100%) no longer have heavy periods<br>• 75 to 100 (75% to 100%) have less pain	• Your period will stop. Out of 100 women, 93 to 100 (93% to 100%) no longer have pain.
No. If you are close to menopause, your periods may become irregular.	Some women have less pain and bleeding. More research is needed.					

What are your thoughts?

Will the fibroids go away or get smaller (in size)?

Watch and wait	Medicine without hormones	Medicine with hormones	Embolization (blocking blood flow)	Endometrial ablation (destroy lining of uterus)	Myomectomy (surgery to remove fibroids)	Hysterectomy (surgery to remove uterus)
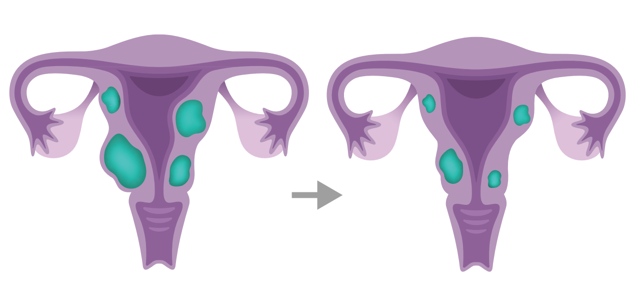	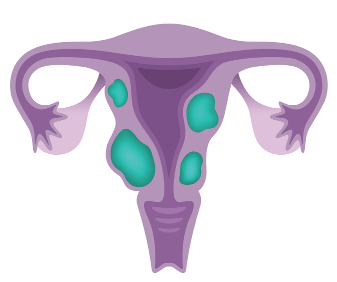	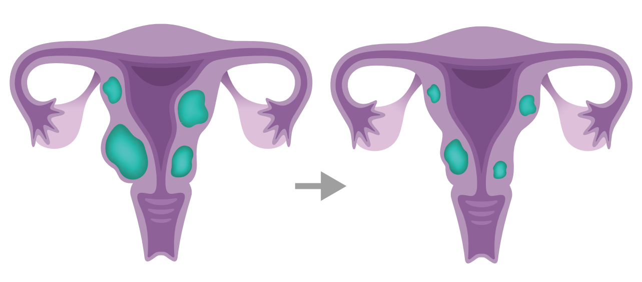	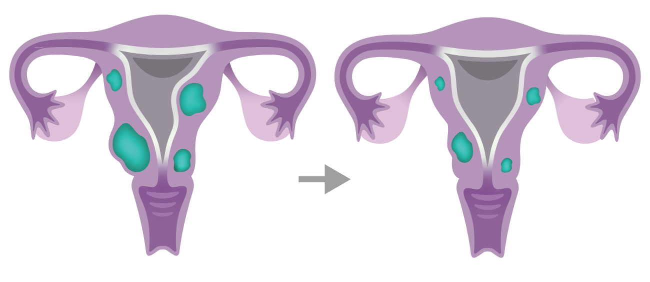	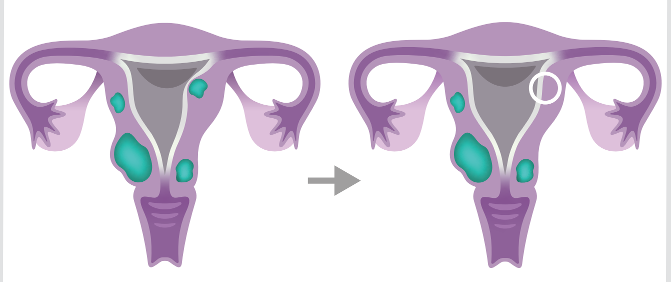	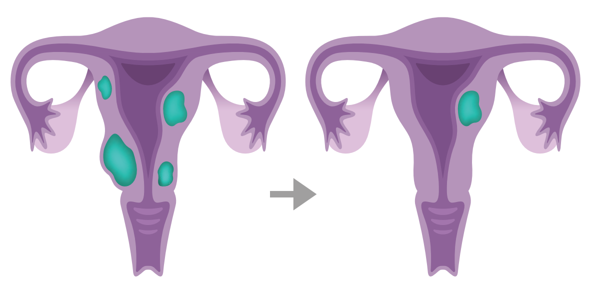	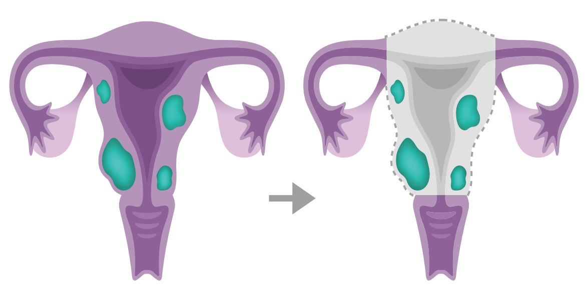
Your fibroids may get smaller with menopause and cause less problems. If you are younger, fibroids may get bigger.	No.	After leuprolide injections, fibroids may get smaller. They grow back when you stop. Other hormone medicines may not help.	Your fibroids may get smaller.	Research is limited. Very small fibroids near the uterus lining may go away when the uterus lining is removed.	Most fibroids can be removed.	Your fibroids will be removed with your uterus.

Is it safe to get pregnant?

Watch and wait	Medicine without hormones	Medicine with hormones	Embolization (blocking blood flow)	Endometrial ablation (destroy lining of uterus)	Myomectomy (surgery to remove fibroids)	Hysterectomy (surgery to remove uterus)
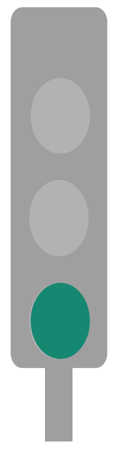	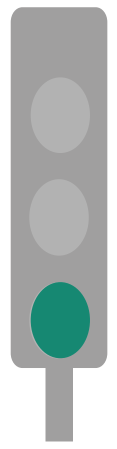	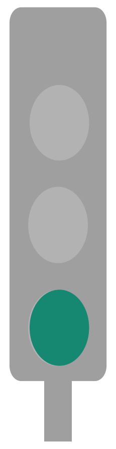	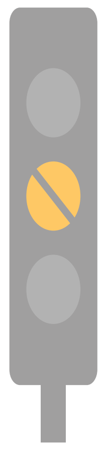	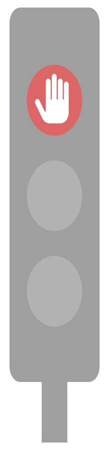	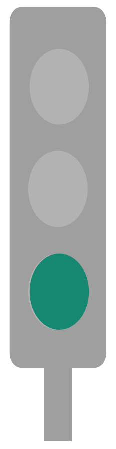	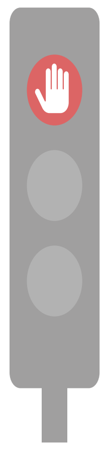
Yes.	Yes.	Yes, but you will need to stop the medicine before trying to get pregnant.	More research is needed to answer this question for this option.	No. There are serious risks to being pregnant. Discuss birth control.	Yes. You may need to deliver by C-section.	No. You will no longer be able to get pregnant.

What are your thoughts?

What are the side effects?

Watch and wait	Medicine without hormones	Medicine with hormones	Embolization (blocking blood flow)	Endometrial ablation (destroy lining of uterus)	Myomectomy (surgery to remove fibroids)	Hysterectomy (surgery to remove uterus)
	Out of 100 women, up to:	Out of 100 women, up to:	For a short time, out of 100 women, up to:	For a short time, out of 100 women, up to:	Pain and nausea or throwing up due to anesthesia are common after surgery.	Pain and nausea or throwing up due to anesthesia are common after surgery. If your ovaries are also removed, you will go into menopause.
• There are no side effects.	• 60 (60%) get headaches, mainly with tranexamic acid	• 71 (71%) get hot flashes, mainly with leuprolide injections	• 89 (89%) have pain	• 72 (72%) get cramps		
	• 20 (20%) get pain in their stomach	• 70 (70%) have irregular bleeding	• 64 (64%) get nauseous	• 36 (36%) get nauseous or throw up due to anesthesia		
	• 15 (15%) get nauseous	• 37 (37%) get headaches	• 21 (21%) have vaginal discharge	• 10 (10%) have pain when peeing		

What are the more serious risks?

Watch and wait	Medicine without hormones	Medicine with hormones	Embolization (blocking blood flow)	Endometrial ablation (destroy lining of uterus)	Myomectomy (surgery to remove fibroids)	Hysterectomy (surgery to remove uterus)
		Out of 100 women with an IUD:	Out of 100 women:	Out of 100 women, up to:	Risks depend on how the myomectomy is done. Out of 100 women, up to:	Risks depend on how the hysterectomy is done. Out of 100 women, up to:
• There are no serious risks	• Less than 1 out of 100 women (1%) have a stroke, blood clot, stomach bleed, or ulcer.	• 23 (23%) do not get better or have problems with it by 1 year	• 24 (24%) need more surgery by 2 years	• 12 (12%) need more surgery by 2 years	• 7 (7%) need more surgery by 2 years	• 12 (12%) get an infection
		• It falls out in up to 10 (10%)	• 3 (3%) get an infection	• 6 (6%) get an infection	• 3 (3%) need a blood transfusion	• 7 (7%) need a blood transfusion
		• Less than 1 (1%) get infection or injury	• Less than 1 (1%) get a blood clot	• 5 (5%) get an injury to their uterus	• Less than 1 (1%) need a hysterectomy during surgery	• 6 (6%) get a urinary system injury

What are your thoughts?
